# Outcomes and characteristics of patients on protease inhibitors at a tertiary level antiretroviral clinic

**DOI:** 10.4102/sajhivmed.v24i1.1536

**Published:** 2023-12-21

**Authors:** Michele Perks, Denasha L. Reddy, Francois Venter

**Affiliations:** 1 Department of Internal Medicine, Faculty of Health Sciences, University of the Witwatersrand, Johannesburg, South Africa; 2 Department of Internal Medicine and Infectious Diseases, Faculty of Health Sciences, University of the Witwatersrand, Johannesburg, South Africa; 3 Wits Ezintsha, Faculty of Health Sciences, University of the Witwatersrand, Johannesburg, South Africa

**Keywords:** HIV, NRTI recycling, protease inhibitors, antiretrovirals, South Africa

## Abstract

**Background:**

Protease inhibitors (PIs) have been recommended as World Health Organization second-line antiretroviral therapy (ART) for low- to middle-income countries for two decades. As dolutegravir-based regimens have become widely available, the future role of PIs is uncertain.

**Objectives:**

To describe the characteristics of patients on PI-based ART (in first-line and second-line regimens), double-boosted protease inhibitors (DBPI) and patients who received recycled nucleoside reverse transcriptase inhibitors (NRTI) in second-line regimens at a tertiary level ART clinic.

**Method:**

We conducted a descriptive retrospective record review of adult patients on PI-based ART who attended Nthabiseng Adult Infectious Diseases Clinic at Chris Hani Baragwanath Academic Hospital in Soweto, South Africa, between January 2021 and April 2022.

**Results:**

Of the 900 patients sampled, 543 (60.3%) were female, the median age was 45 and 703 (79.1%) had viral loads (VL) below 1000 copies/mL. In contrast, 21 (58.3%) of 36 vertically infected patients had VLs below 1000 copies/mL. Thirty-seven (4.1%) patients were on DBPIs. The commonest reason for DBPI use in 24 (64.9%) patients was drug resistance test (DRT)-guided switch after virological failure. Forty-nine (5.4%) patients were on recycled NRTIs with no DRT, and 24 (2.6%) patients were on NRTIs to which there was documented resistance. Outcomes for these patients were similar to the total sample.

**Conclusion:**

PIs have long been a cornerstone of second-line ART. This study demonstrates the real-world utility of PIs, as well as their disadvantages. There was no difference in the outcomes of patients who received recycled NRTIs in second-line regimens.

**What this study adds:** Our study offers a historical look at the use of PIs in South Africa and supports NRTI recycling.

## Introduction

South Africa (SA) has the greatest burden of HIV and manages the largest antiretroviral therapy (ART) programme globally.^1,2^ Approximately 62% of people living with HIV or AIDS (PLWHA) in SA are currently on ART, with 86% of them being virally suppressed.^1^ There has been improved access to viral load (VL) monitoring, earlier detection of virological failure (VF) and more appropriate transition from first-line to second-line and third-line therapies, as these regimens have evolved to be less toxic and more potent.^3,4^ Despite these achievements, SA is still falling short of the The Joint United Nations Programme on HIV/AIDS 90-90-90 targets, which aimed to have 90% of PLWHA on ART being virally suppressed by 2020.^5^

A boosted protease inhibitor (PI) and two nucleoside reverse transcriptase inhibitor (NRTI) combination has long been the recommended second-line ART regimen for low- to middle-income countries (LMICs) until fairly recently.^6^ The SA National Department of Health 2023 guidelines now recommend the fixed-dose combination of tenofovir (TDF), lamivudine (3TC) and dolutegravir (DTG), known as tenofovir/lamivuidne/dolutegravir (TLD), in both first-line and second-line ART regimens.^7^ They recommend that patients on TDF, emtricitabine (FTC) and efavirenz (EFV) be switched to TLD regardless of VL result, and patients on PI-based second-line regimens with a VL less than 1000 copies/mL can be switched to TLD, regardless of previous NRTI use or resistance patterns.^7,8^ Dolutegravir is better tolerated than EFV-based regimens with a higher barrier to resistance and has shown better safety and efficacy as compared to boosted PIs.^6,9^

These recommendations are based, most notably, on trials such as EARNEST (Europe–Africa Research Network for Evaluation of Second-Line Therapy),^10^ which hypothesised that replacing the NRTI class with an integrase inhibitor in second-line treatment, thereby introducing two new drugs, would be superior to standardised NRTI+PI regimens. However, they found no benefit over the standard regimen, suggesting that despite predicted and proven genotypic NRTI resistance, even recycled NRTIs played an important role in the efficacy of the regimen.^10,11,12,13^ Thereafter, more recent trials such as NADIA,^14^ VISEND^15^ and ARTIST^16^ took the question of NRTI recycling in second-line regimens a step further and assessed its utility with DTG-based second-line treatment. They demonstrated that TDF and 3TC/FTC could be successfully recycled in second-line treatment, allowing the fixed-dose combination of TLD to be used in both first-line and second-line regimens.

In this new DTG era, the future role of PIs is uncertain. Thus far, DTG resistance remains very rare. However, it has been demonstrated in clinical trials and a few clinical cases, more so in treatment-experienced patients.^17,18,19^ The vast majority of the evidence behind the current guidelines is from clinical trials; therefore, the real-world clinical implications are yet to be made clear. The SA ART guidelines state that if a patient develops confirmed VF after two years on DTG-based ART, especially those who have had ART exposure prior to TLD, it should be discussed with an HIV expert for possible genotypic testing, with the potential need for PI-based regimens.^7^ Therefore, PI-based regimens will likely remain relevant and important.

### Aims and objectives

This study aimed to describe the demographic, clinical and laboratory characteristics and outcomes of adults on PI-based first-line and second-line ART, including patients on double-boosted PIs (DBPI), at a tertiary level hospital in Johannesburg, SA. Additional aims were to assess the reasons for patients switching to PI-based therapy, drug changes on PI-based ART and the types and prevalence of side effects and toxicity related to PI-based therapy. Furthermore, the study aimed to identify and describe a subset of patients who received recycled NRTIs in second-line treatment regimens and to describe the virological and immunological outcomes of this group. The study may contribute to the knowledge about characteristics and outcomes of treatment-experienced patients on PI-based regimens in real-world clinic settings in LMICs. This is still important, despite the new DTG era, because PIs have long been a potent cornerstone of HIV management, and with the true clinical durability of DTG yet to be fully elucidated, PIs are likely to still play an important role in the management of HIV.

## Methods

The study design was a retrospective descriptive record review. The study site was Nthabiseng Infectious Diseases Clinic at Chris Hani Baragwanath Academic Hospital, which is at a large public tertiary hospital in Johannesburg, Gauteng. The hospital serves the large urban population of Soweto and surrounding areas.

A list of patients on first-line or second-line PI-based ART was obtained from the data capturers working at the clinic. Any adult patients (> 18 years old) on first-line or second-line PI-based ART (either a PI and two NRTIs or DBPI) with available clinic records were included in the study. Exclusion criteria were age less than 18 years old and the absence of a clinic record. The patients were randomly sampled from the list, and records were drawn with assistance from the clinic clerks and with permission of the head of infectious diseases and internal medicine and hospital management at Nthabiseng Infectious Diseases Clinic at Chris Hani Baragwanath Academic Hospital. Data collection took place between January 2021 and April 2022.

The records were reviewed privately by the primary author on site at the clinic and returned the same day to the clerks. Data were captured on a standardised data collection instrument by the primary author. Each patient was assigned a number so that no personal patient identifying information, such as name or hospital number, was captured during data collection. With permission of the head of the clinic, each file was marked so that duplicate reviews of the same file would be avoided.

The primary author of the study was the only record reviewer and data handler; therefore, unfortunately, they could not be blinded, and interrater reliability was not determined. The primary author is a medical doctor who has worked within the study site and is familiar with the standardised note-keeping template that the clinic employs to aid uniform record-keeping.

The variables of primary interest included age, sex, race, current PI-based ART regimen, previous ART exposure, VL and CD4 count. Other variables of interest that were collected included reasons for change in ART regimen, date of initiation of ART, date of initiation of PI-based therapy, total duration of PI-exposure, baseline and latest total cholesterol and triglyceride values, haemoglobin before and after (within one year) PI switch, hepatitis B surface antigen status, indication in the clinic notes that the patient acquired HIV through vertical infection, and drug resistance test (DRT) results. Although all data from retrospective record review is inherently prone to bias due to high levels of missing data, inaccuracy of record-keeping and data abstraction, the primary variables of interest were deemed more reliable due to uniformity of the variable in question and less room for subjective interpretation of data points.

Patients were classified according to the PI regimen they were on at the time of data collection. All previous ART regimens were recorded. If a patient was exposed to the same NRTI in first-line and in second-line regimens, they were highlighted as having NRTI recycling. Patients who were on a PI-based first-line regimen without evidence of VF at the time of commencing the PI were not included in the NRTI recycling group. In addition, recycling of 3TC and FTC in second-line regimens was not included in the NRTI recycling group, as there is broad agreement around the utility of this recycling due to the fitness cost to the virus and partial restoration of susceptibility to other NRTIs associated with the M184V resistance mutation to 3TC/FTC. Therefore, we focused on the recycling of the other NRTIs.^6^ If DRT was done at the time of switch to a PI-based regimen, these results were recorded, and if the patient was shown on DRT to have susceptibility to the recycled NRTI in question, then they were excluded from the recycled NRTI group. Drug resistance test results were compared to the current ART regimen, as in many patients DRT was done many years ago, and regimens had subsequently changed. A subset of patients was identified as having documented resistance on DRT to NRTIs that they were currently on.

Starting dates of different regimens were recorded by month and year. If only the year was available, the month was rounded off to the first of June of that year. Viral load results that were recorded as ‘lower than detectable’ in clinic records were captured as 50 copies/mL in the data collection as the National Health Laboratory Services, where laboratory results were obtained from, generally use a detectable threshold of 50 copies/mL. Percentages were calculated with the denominator based on the total number of patients for which the answer to any one variable was known, that is, missing data points were excluded from calculating percentages.

### Statistical analysis

Demographics, ART regimens, side effects and laboratory characteristics were summarised using frequencies for categorical variables and means for normally distributed data, or medians for not normally distributed data. Microsoft Excel was used for the descriptive portion of the data analysis. In addition, we used Stata 18 to perform a chi-squared test to determine if there is an association between different VL categories across different groups.

### Ethical considerations

Ethics approval was granted by the Human Research Ethics Committee (Medical) of the University of the Witwatersrand, Johannesburg (ref. no. M200612). Confidentiality of patients was maintained by each record being given a study number, and no personal identifying information was recorded. The data were captured on site at the clinic in a private room by the primary author, and the records were returned to the clinic clerks on the same day.

## Results

### Study sample and demographics

The total sample included 900 patients. Five hundred and forty-three (60.3%) were female, and the median age was 45 years. Eight hundred and ninety-one (99.0%) of the patients were Black, 7 (0.8%) were mixed-race, and 2 (0.2%) were White. The median time on PI was 6 years. The longest period a single patient was on a PI was 18 years. The ART regimen that was used prior to a PI regimen was unknown in 156 (17.3%) patients. Tenofovir/emtricitabine and EFV was the most commonly used initial ART regimen in 361 (48.5%) patients, and stavudine (D4T)/3TC and EFV was the second most commonly used initial regimen in 275 (37.0%) patients. One hundred and thirty-three patients (17.9%) were on more than one ART regimen prior to the PI regimen. The majority of changes in the initial regimen were in the NRTI backbone in 114 cases, whereas only 14 patients changed from one non-nucleoside reverse transcriptase inhibitor (NNRTI) to another.

The median VL was 56 copies/mL, with 79.1% having a VL less than 1000 copies/mL. The median CD4 count was 460 cells/µL, with 84.0% being over 200 cells/µL. Tenofovir, FTC and EFV was the most-used first-line regimen in 48.5%. The standard empiric second-line PI regimen (zidovudine [AZT], 3TC and Lopinavir/ritonavir [LPVr]) was being used in 37.7% of patients at the time of data collection. The second most used PI regimen, at 17.7% of patients, was TDF, FTC and LPVr (Table 1).

**TABLE 1:** Characteristics of patients on protease inhibitor-based antiretroviral therapy.

Current PI regimen	All (*N* = 900; 100%)			LPVr-based regimens (*n* = 572/*N* = 900; 63.6%)			ATVr-based regimens (*n* = 301/*N* = 900; 33.4%)			ATV/LPVr (DBPI) (*n* = 27/*N* = 900; 3.0%)		
	*n*	%	Median	*n*	%	Median	*n*	%	Median	*n*	%	Median
**Age (years)**	-	-	45	-	-	45	-	-	44	-	-	45
**Gender**												
Female	543	60.3	-	345	60.3	-	182	60.5	-	16	59.3	-
Male	357	39.7	-	227	39.7	-	119	39.5	-	11	40.7	-
**Hepatitis B status**												
sAg positive	45	5.0	-	32	5.6	-	13	4.3	-	–	–	-
sAg negative	855	95.0	-	540	94.4	-	288	95.7	-	27	100	-
**CD4 Count (cells/µL)**	-	-	459.5	-	-	-	-	-	443	-	-	-
Unknown	17	1.9	-	13	2.3	470	4	1.3	-	–	–	420
≤ 50	29	3.3	-	15	2.7	-	12	4.0	-	2	7.4	-
51–200	112	12.8	-	75	13.4	-	36	12.1	-	1	3.7	-
> 200	742	83.9	-	469	83.9	-	249	83.9	-	24	88.9	-
**Viral load (copies/mL)**	-	-	56	-	-	51	-	-	52	-	-	88
Unknown	11	1.2	-	11	1.9	-	-	-	-	-	-	-
≤ 50	434	48.8	-	280	49.9	-	146	48.5	-	8	29.6	-
51–999	269	30.3	-	168	29.9	-	87	28.9	-	14	51.9	-
1000–9999	76	8.5	-	42	7.5	-	32	10.6	-	2	7.4	-
≥ 10 000	110	12.4	-	71	12.7	-	36	12.0	-	3	11.1	-

Note: All: Time on PI (months) = 73; LPVr-based regimens: Time on PI (months) = 79.5; ATVr-based regiments: Time on PI (months) = 67; ATV/LPVr (DBPI): Time on PI (months) = 56.

PI, protease inhibitors; ATVr, Atazanavir/ritonavir; DBPI, Double-boosted protease inhibitor; LPVr, Lopinavir/ritonavir; sAg, Surface antigen.

### Vertically transmitted group

Thirty-six patients were identified through indication in the clinic records as having acquired HIV through vertical transmission at birth. The average age of this group was 22 years, with the oldest patient being 29 years old. Only 21 (58.3%) patients in this group had a VL lower than 1000 copies/mL, and only 7 (19.4%) had VLs lower than 50 copies/mL. Half (50%) of these patients were on TDF, FTC and atazanavir/ritonavir (ATVr). Patients were preferentially put on this regimen with the rationale that the decreased pill burden and better side effect profile may improve adherence. However, in the group on TDF, FTC and ATVr, 10 (55.6%) had VLs less than 1000 copies/mL, and 4 (22.2%) had VLs less than 50 copies/mL.

### Reasons for switch to protease inhibitor-based regimens

**FIGURE 1:**
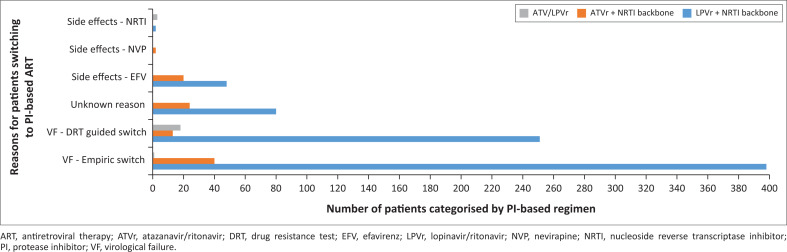
Reasons for patients switching to initial protease inhibitor-based antiretroviral therapy from first-line regimens.

Fifty-five point two per cent of patients had VF and were switched to an empiric second-line PI-based regimen, without DRT, as per the standard protocol at that time. A DRT is not generally available outside of second-line failures in SA, but tertiary hospitals have expanded access and, hence, clinicians at the clinic could order limited testing. Thirty-five point four per cent of patients had VF with DRT, and therefore a guided switch and 8.2% were switched to PI-based regimens due to intolerable side effects to first-line drugs, with no evidence of VF at that time (Figure 1).

### Types and prevalence of side effects

**FIGURE 2:**
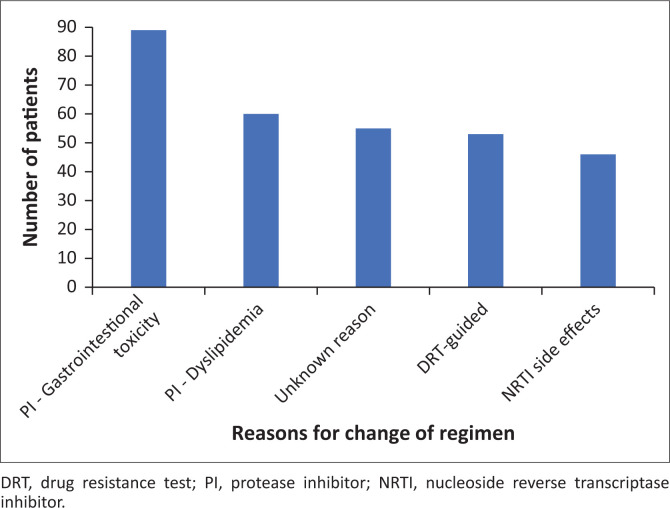
Most common reasons for drug substitution in patients on protease inhibitor-containing regimens.

The most prevalent documented PI side effects were gastrointestinal symptoms and dyslipidemia secondary to LPVr, resulting in 151 (17.9%) patients switching to ATVr. The most common side effects of first-line ART were drug-induced liver injury (2.5%) and gynaecomastia (2.4%), both due to EFV. In terms of NRTI side effects, pure red cell aplasia (PRCA) was documented in 11 patients, three during a first-line NNRTI-regimen and eight while on a PI-based regimen. Eighteen (2.1%) patients developed renal dysfunction to such a degree that it necessitated changing TDF to another NRTI. It was not clearly documented in the records if the renal dysfunction was secondary to TDF itself or if there were other causes of renal dysfunction. Three patients were noted to have developed anaemia secondary to AZT, while eight patients developed anaemia from other causes and required AZT to be stopped (Figure 2).

### Prevalence of hepatitis B

There were 44 (4.9%) patients with chronic hepatitis B in the sample. The most common regimen used in this group, for 21 (47.7%) patients, was AZT, 3TC and LPVr, with the addition of TDF as a third NRTI for hepatitis B coverage. This regimen was used to cover for possible resistance to TDF as all these patients were exposed to TDF, FTC and EFV in first-line ART. The virological outcomes of this group were similar to the total group, with 34 (77.3%) patients having a VL of less than 1000 copies/mL (Table 1).

### Double-boosted protease inhibitor group

A total of 37 (4.1%) patients were on a DBPI regimen at some point, with 27 (3.0%) of these patients on DBPI at the time of data collection. All patients were on a combination of ATVr and LPVr, with only one patient who had 3TC added to the regimen. Fifteen patients (1.7%) were found to have been on DBPI for the duration of their second-line regimen, with an average time on DBPI of 4.6 years and a maximum of 7.3 years. Of these 37 patients, 17 (45.9%) patients were started on DBPI based on the initial DRT, done when the patient failed first-line ART. Seven (18.9%) patients were started on DBPI after a DRT was done due to VF on empiric second-line treatment. Ten (27.0%) patients had side effects to NRTIs, the majority of which were 3TC-induced PRCA, in either initial first-line or empiric second-line treatment, which prevented the use of NRTIs.

Seventeen (45.9%) patients had DRTs showing some degree of resistance to all available NRTIs. Three patients had sensitivity to AZT only; however, they had anaemia, which contraindicated its use at that time. Four patients had no documented reason for DBPI, and two patients had unavailable DRT results. Of the patients currently on DBPI, 22 (81.5%) patients had a VL of less than 1000 copies/mL. Three (8.1%) of the patients who were ever on DBPI were taken off DBPI regimens due to dyslipidemia, and this is comparable to the total sample group in which 60 (7.1%) patients were switched from LPVr to ATVr due to dyslipidaemia (Figure 2).

### Nucleoside reverse transcriptase inhibitors recycled group

A total of 721 (80.1%) patients were on PI-based second-line treatment for VF. Of these, 156 (17.3%) did not have any record of their first-line antiretroviral exposure. Out of the remaining 565 (62.8%) patients, 146 (16.2%) were at some point on an NRTI (excluding 3TC and FTC) in second-line that they had already been exposed to in their first-line regimen. In addition, due to the shared resistance mutations between TDF and abacavir (ABC), any switch from one of these to the other was also considered as NRTI recycling. Forty-nine (5.4%) of these patients had recycled NRTIs without any DRT to prove sensitivity to the NRTI. Forty (4.4%) of the 49 patients were currently on this NRTI at the time of data collection.

Tenofovir was the most commonly recycled NRTI for 20 (40.8%) patients. Fifteen (30.6%) patients were exposed to TDF in their first-line regimen and then put on an ABC-containing PI regimen at some point after VF. Renal dysfunction, along with a contraindication or intolerability to AZT use, may have been a contributing factor to this observation. Reasons for NRTI recycling included side effects or contraindications to other non-recycled NRTIs with no other available NRTI, the first-line ART history not being known to the clinician resulting in inadvertent recycling and healthcare workers not being aware of the shared resistance mutations between TDF and ABC. Five patients were put onto recycled NRTIs due to a drug shortage of AZT, with no other option for NRTI. Forty (81.6%) patients had a VL below 1000 copies/mL, and 24 (49.0%) patients had undetectable VLs.

### Documented resistance to nucleoside reverse transcriptase inhibitors on current regimen group

Twenty-four patients were found to currently be on PI-based regimens that included an NRTI (excluding 3TC/FTC) to which they had documented resistance on DRT. Of the 24 patients, 19 had resistance to a recycled NRTI, with five who had resistance to an NRTI that they had never been exposed to. Half of the patients only had low-level resistance to the NRTI, eight had intermediate-level resistance, and four had high-level resistance. Tenofovir was the most commonly used NRTI in this group with documented resistance – with nine patients having low-level resistance, four with intermediate-level resistance and three with high-level resistance. Twenty-one (87.5%) patients had a VL of less than 1000 copies/mL, and 13 (54.2%) were fully suppressed on their regimen.

**FIGURE 3:**
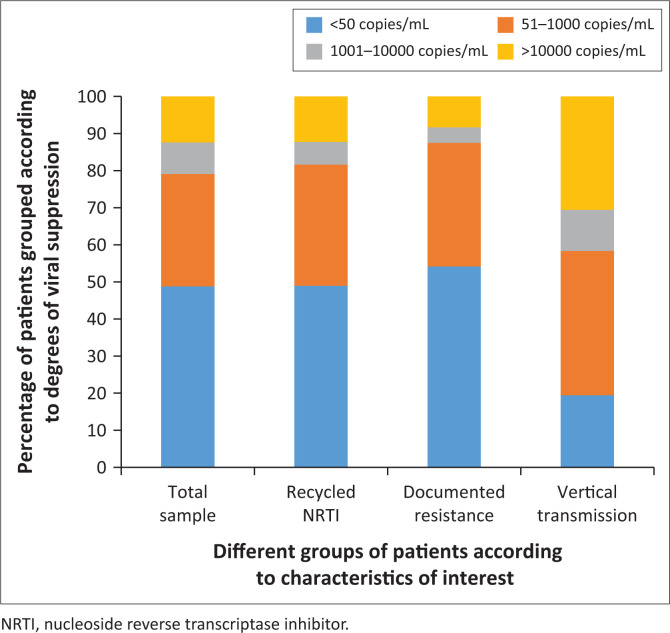
Viral suppression across different groups.

Figure 3 shows a comparison of the VLs across different groups. We performed a chi-squared test of association between VL categories and the groups – NRTI recycling, documented resistance and vertical transmission – which showed a significant association with a *P*-value of 0.047.

## Discussion

Among the 900 patients on PI-based ART regimens, the median VL was 56 copies/mL, with 79.1% of patients having a VL less than 1000 copies/mL and 434 (48.8%) with VLs lower than the detectable limit. This is less than the reported national average of 86% of patients on ART being virally suppressed (defined as a VL less than 1000 copies/mL by UNAIDS).^1^ However, a possible reason for this is that as it is a tertiary level clinic, it receives a disproportionate number of complicated patients from local clinics and secondary hospitals who require higher levels of care for problems such as suspected VF on second-line ART. In addition, patients who are doing well on second-line ART are often down-referred to lower levels of care; however, it is equally possible that patients who were unwell may have died or were lost to follow-up.

This study offers an interesting historical look into ART use in SA, as the median time on PI was 6 years, and the longest time a single patient was on a PI-based regimen was 18 years. In addition, 37.0% of patients were on D4T, 3TC and EFV as an initial first-line regimen prior to the availability of TDF in SA in 2007. Sixty-five (6.4%) patients used didanosine (DDI) in initial second-line PI-based regimens due to this being the initial recommendation for second-line regimen NRTI components before being replaced with recycled 3TC. Thirty-five per cent of patients had a DRT-guided switch from first-line to second-line, which is higher than one would expect as it is recommended that an empiric switch be made to second-line ART in LMICs with poor access to expensive DRT.^6,7^ Possible contributing reasons for this observation are that patients who had DRT-guided switches were found to have had extensive prior NRTI use or unknown first-line regimens, and DRT was possibly performed as it was expected to assist with choosing the best PI-based second-line regimen. In addition, the average time on a PI regimen was 6 years, which would date the time of switch well before DTG and favourable NRTI recycling recommendations when second-line and third-line options were more limited. However, with the results of recent studies showing the retained utility of NRTIs in second-line (either PI-based or DTG-based second-line) regimens despite previous use of the same NRTI in first-line, the evidence to support using DRT in this population is poor even in the face of extensive previous NRTI use.^8,10^ This study, although retrospective and observational, demonstrates that empiric second-line switches have shown comparable virological outcomes with DRT-guided second-line switches.

Thirty-six patients in this adult population acquired HIV through vertical transmission. This group had less viral suppression as compared to the total sample, with 41.7% having VLs over 1000 copies/mL, almost double that seen in the total sample. Eighteen (50.0%) of these patients were on a regimen of TDF/FTC/ATVr, with the presumed rationale that this regimen may improve adherence due to better tolerability, once-daily dosing, and a lower pill burden. However, only 10 (55.5%) of these patients had a VL below 1000 copies/mL. Therefore, despite thinking this regimen would improve adherence, the VLs for this group are similar to those in this group on other PI-based regimens. This may indicate ongoing adherence issues. However, true resistance would need to be considered as these patients may be particularly treatment-experienced as compared to other groups. This observation underscores the importance of adherence and that despite providing patients with simpler and better tolerated regimens, adherence remains a major factor in ensuring good outcomes. This observation is particularly relevant in the new era of DTG-based fixed-dose combination where it cannot be presumed to be a silver bullet. Optimal care and surveillance need to be maintained to ensure the durability of DTG and the proper care of our patients.

Forty-nine patients were identified as having recycled NRTI in their second-line regimen. Eighty-one point six of those with recycled NRTIs had a VL of less than 1000 copies/mL, compared to 79.1% in the total sample, showing comparable viral suppression, suggesting that despite NRTI recycling, patients had similar virological outcomes. Similarly, 24 patients were identified to currently be on an NRTI, which they previously had documented resistance to on DRT, and 87.5% of these patients had a VL of less than 1000 copies/mL (Figure 3). These findings are in keeping with results from trials showing that despite extensive NRTI use and documented resistance, their utility has been shown to be retained when used with boosted PI.^10,11,12,13^ The reasons for the observed recycling of NRTIs in this study, prior to the current acceptance of its utility, are numerous. These may include poor knowledge of shared resistance patterns between NRTIs, accidental recycling due to lack of knowledge of previous ART regimens or contraindications such as renal failure or anaemia preventing the use of available NRTIs, leaving no other viable option other than to recycle NRTIs. However, the study also demonstrates this clinic’s use of DBPI regimens, which were utilised in some cases of extensive NRTI resistance or class side effects. With the availability of a wider range of effective drugs with better tolerability and the new knowledge of NRTI recycling there is no real utility for DBPI regimens anymore.

Five percent of the total sample were hepatitis B surface antigen-positive, which is comparable to the estimated prevalence of co-infection of HIV and hepatitis B in SA of 5% – 9%.^20,21^ Seventy-three point three percent of these patients at the time of data collection were still on a regimen of AZT/3TC/LPVr with additional TDF to treat hepatitis B, due to the presumed TDF resistance from first-line regimens. Although the 2019 SA ART guidelines had by this time recognised the findings of the EARNEST trial and recommended that in hepatitis B surface antigen-positive patients, additional AZT was not necessary, it was not fully reflected in the results of the study at that time.

The retrospective nature of this study, along with the referral bias inherent in tertiary services, resulted in many limitations, which included high dependency on the accuracy of recorded data in clinic records and a high degree of missing data, which varied greatly depending on the variable in question. The authors acknowledge that the strength of the study could have been improved with the addition of more than one data abstractor, abstractor monitoring and determination of interrater reliability.

## Conclusion

This study examined the records of a real-world population of PLWHA, many of whom were treatment-experienced, on PI-based regimens. It demonstrated the utility and potency of PI use, the use of PI regimens in patients with side effects to first-line agents, as well as DBPI regimens in those with class side effects to NRTIs or widespread NRTI resistance. The challenges of PI use are clearly demonstrated with sizeable numbers of patients switching from LPVr for dyslipidaemia and gastrointestinal side effects. A small subset of patients who had NRTI recycling on PI-based second-line treatment for VF was identified, and in keeping with studies looking at NRTI recycling, these patients’ outcomes were comparable to those who did not have NRTI recycling. This supports recent recommendations to recycle NRTIs from first-line to second-line ART regimens.
